# Impact of matrix systems on proximal contact tightness and surface geometry in class II direct composite restoration in-vitro

**DOI:** 10.1186/s12903-023-03222-5

**Published:** 2023-08-02

**Authors:** Zeinab Omar Tolba, Ezzat Oraby, Possy Moustafa Abd El Aziz

**Affiliations:** 1 Conservative Dentistry Department, Faculty of Dentistry, Cairo University, Cairo, Egypt; 2 National Institute of Standards (Engineering and Surface Metrology), Giza, Egypt

**Keywords:** Circumferential matrix, Contact tightness, Pre-contoured matrix, Restoration proximal contour, Sectional matrix

## Abstract

**Background:**

Poor contact tightness and contour in class II composite restorations are significant problems in clinical practice. They affect occlusal stability and periodontal health. The aim of this study was to evaluate proximal contact tightness and contour established after completing class II direct composite restorations using two pre-contoured matrix systems.

**Methods:**

Standardized mesio-occlusal cavities were prepared in twenty typodont lower right first permanent molar teeth. Prepared teeth were randomly divided into two groups according to matrix system: Group 1, Sectional matrix system with a separation ring (Palodent V3); and Group 2, Circumferential matrix system with integrated tightener (Palodent 360). Contact tightness was evaluated using universal testing machine. Area, depth and curvature radius of proximal surface concavity in the restoration were evaluated using contact stylus profilometer. T-test was used for comparison between groups.

**Results:**

Sectional matrix showed higher contact tightness than circumferential matrix system. The results of proximal surface concavity in the restoration showed significantly higher area and depth of concavity with lower radius of curvature in circumferential matrix compared to sectional matrix.

**Conclusions:**

The use of separation ring with sectional matrix provides superior contact tightness compared to circumferential matrix. However, both matrix systems presented some deficiency regarding proximal contour of direct class II resin composite restoration.

## Background

Clinicians often find it challenging to perform posterior class II direct composite restoration in an efficient and predictable manner. The key to success lies in achieving a well-sealed restoration with good contact and contours [1]. Both, inter- and intra-individual variability exist in proximal contact [2, 3]. A loose proximal tooth contact would permit food impaction and cause subsequent tooth migration, dental caries, or periodontal disease. Whereas, excessively tight proximal contact causes patient discomfort and undesirable tooth movement. Additionally, too tight contact hampers pass of dental floss through the contact area resulting in trauma to the periodontium. Thus, development of techniques that guarantee adequate proximal contact and contour is highly recommended. Several techniques were developed, such as increasing the viscosity of restorative composites, use of a separation ring system, and contoured matrix. Studies have found that the matrix system used may exert a more significant effect than the consistency of composite resin [3, 4].

Sectional matrix techniques using separating rings are referred to as gold standard due to predictable establishment of contact areas and strong proximal contact tightness [5]. However, they have been shown to result in surface concavity of the restoration at the contact area. Meanwhile, the flat circumferential matrices resulted in an inferior morphological contact with reduced contact tightness [5].

A new circumferential matrix system, Palodent 360, specifically designed for multi-surface restorations, was recently introduced. Its bands are supplied as pre-contoured thin bands (0.032 mm thin) that are similar to sectional matrix bands. So; these bands enable re-establishment of proper anatomical proximal contact and contour, with proper contact tightness. Unlike retained circumferential matrices, the new Palodent 360 matrix system is placed as a single component with an integrated tightening mechanism for better accessibility and efficient workflow.

Nevertheless, no solid scientific evidence to support this newly introduced technique is available, hence the need for more studies that evaluate its effects are needed. Therefore, the aim of the present study was to evaluate the proximal contact tightness and contour established after completing class II direct composite restorations using two pre-contoured matrix systems. The null hypothesis is that the pre-contoured circumferential matrix will lead to similar contact tightness and contour compared to the gold standard sectional matrix.

## Methods

### Ethical approval and samples preparation

Ethical approval was granted by the Research Ethics Committee of Faculty of Dentistry, Cairo University (number:171022) in view of the in-vitro nature of the study.

Twenty Nissin typodont lower right first permanent molar teeth were included in this study. Standardized mesio-occlusal cavities were prepared using diamond burs in a high speed hand-piece: with dimensions of 4 × 3 × 1.5 mm (buccolingual, occlusogingival, mesiodistal, respectively), and 1.5 mm occlusopulpal. Prepared teeth were then randomly divided into two groups according to the matrix system used of 10 teeth each. Group 1: Sectional matrix system and a separation ring (Palodent V3, Dentsply Sirona, USA); Group 2: Circumferential matrix system with integrated tightener (Palodent 360, Dentsply Sirona, USA). A power analysis was designed to have adequate power to apply a two-sided statistical test of the null hypothesis that there would be no difference between tested groups regarding proximal contact tightness. By adopting an alpha level of (0.05) a beta of (0.2), i.e., power = 80%, and an effect size (d) of (1.40) calculated based on the results of a previous study [6]; the predicted sample size (n) was found to be (20) teeth (i.e. 10 teeth per group). Sample size calculation was performed using G*Power version 3.1.9.7 [7].

### Restorative procedure

**Fig. 1:**
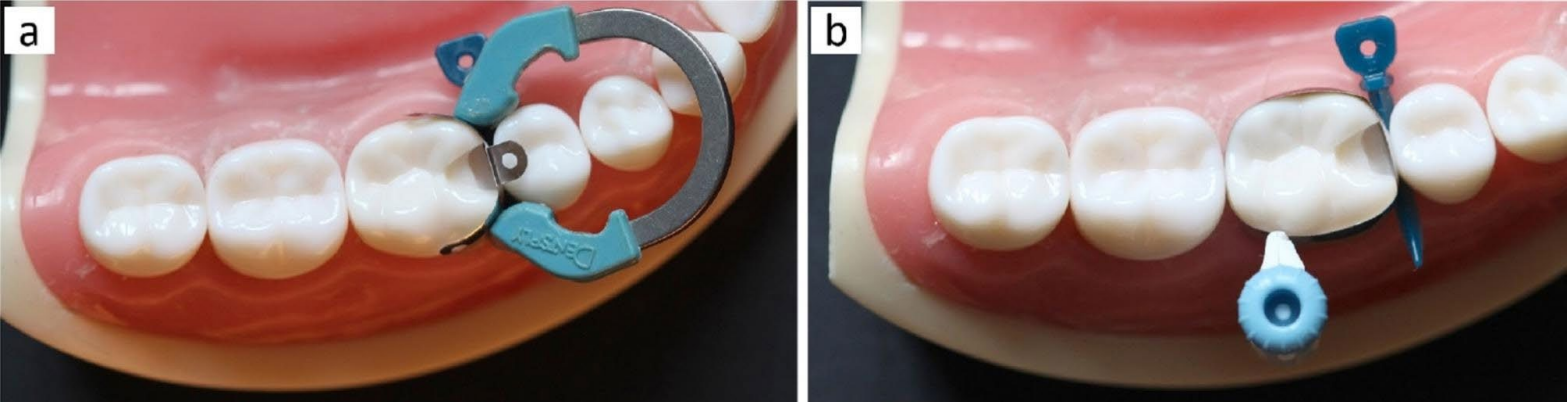
The matrix systems used: (**a**) sectional matrix, (**b**) circumferential matrix

For both groups, the used matrix bands were pre-contoured of 5.5 mm height and 0.032 mm thin. In group 1, sectional matrix band was grasped with pin tweezer and placed at the mesial surface of the prepared cavity, then placing the separation ring interproximally. While in group 2, circumferential matrix was placed around the tooth then the integrated tightener was twisted to tighten the matrix band. In both groups, the gingival margin was secured by anatomic plastic wedge (Palodent V3 Plus Wedge, Dentsply Sirona, USA) as shown in Fig. 1. The bands were not burnished as they were pre-contoured and for standardization. Prior to adhesive procedures, the adaptation of the matrix band at the gingival cavity margin was checked with an explorer.

The adhesive (ALL- BOND UNIVERSAL) as shown in Table 1, was applied according to the manufacturer’s instructions and cured for 10 s using light cure unit with intensity more than 1500mW/cm^2^ (Rainbow, Zhengzhou Kongsin dental, Guangdong, China). Tetric N-Ceram nano-hybrid composite was then placed in three increments. Each layer was separately cured for 20 s. Finally, the restorations were finished and polished using Enhance® finishing and polishing systems (Dentsply Sirona, USA). All the restorative procedures were performed by the same operator.

**Table 1:** The materials used in the study

Material	Composition	Manufacturer	Lot
ALL-BOND UNIVERSAL (Universal Adhesive)	Bisphenol A diglycidylmethacrylate, ethanol, MDP, 2-hydroxyethyl methacrylate, Water, Initiator, pH 3.2	BISCO, Schaumburg, USA	2100001403
Tetric N-Ceram (Nano-hybrid composite)	Dimethacrylates (19–20 wt%); the fillers contain barium glass, ytterbium trifluoride, mixed oxide and copolymers (80-81wt.%). Additives, initiators, stabilizers and pigments are additional contents (˂ 1 wt%). The total content of inorganic fillers is 55–57 vol%. The particle size of inorganic fillers is between 40 and 3000 nm.	Ivoclar Vivadent, Liechtenstein	Z00N7K

### Evaluation of proximal contact tightness

Proximal contact tightness was tested by using universal testing machine (Instron model 3345), the maximum frictional force (N) was exerted on a 0.05 mm-thick stainless steel strip upon withdrawal from the interproximal area. Tensile mode of force was applied at cross-head speed 5 mm/min. The maximum force was enrolled, which was expressed the contact tightness. Data was calculated and recorded using computer software BlueHill universal Instron, England. This technique is similar to Tooth Pressure Meter device which has been previously used in many laboratory and clinical studies [8–11].

### Analysis of Restoration Proximal Contour

The concavity that was observed at the contact area in each restored group was evaluated. The cross-sectional area, depth and radius of curvature of the concavity was measured using contact stylus profilometer (Talysurf- version i60; Metek, UK with software ultra). The profilometer was equipped with a metal probe having a spherical diamond tip of radius 2 microns to assess the surface details. This system was used to analyze the concavity with x - traverse range from 1.5 up to 3 mm and traverse resolution 0.125 microns. The maximum vertical range was 1 mm with vertical resolution 16 nm.

**Fig. 2:**
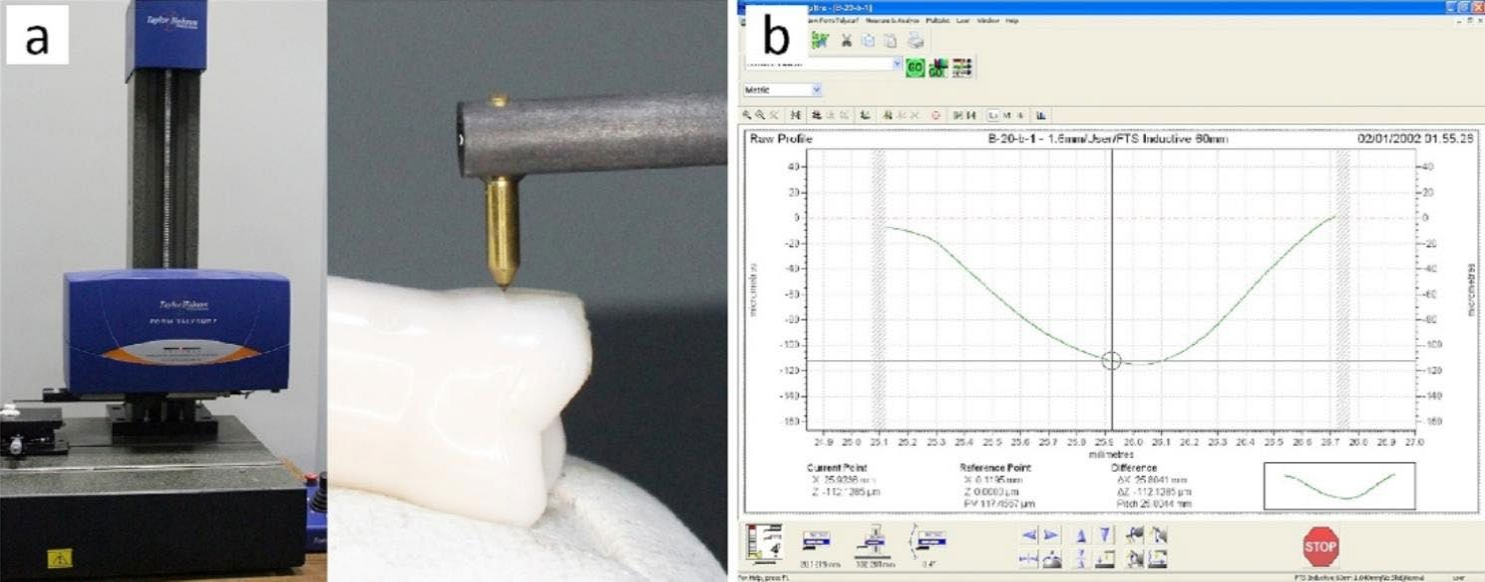
The stylus profilometer system: (**a**) acquisition of data by stylus; (**b**) analysis by software

The stylus system is connected to the computer with a proper software that controls its operation and produces the data of x-z trace to be analyzed. The evaluation trace by the probe was taken across the concavity, making a vertical plane that was perpendicular to the concavity hemi-spherical surface tracing the maximum width of the concavity and passing by the bottom as shown in Fig. 2.

For geometrical characterization of concavity, three parameters were evaluated: (1) cross-sectional area that is bounded by the probe trace (down) and the horizontal line (up), that is the tangent to the opening points of the concavity. (2) the concavity depth and (3) the radius of curvature of the concavity. These parameters would adequately describe the concavity of the restoration. The geometrical analysis was carried out by a developed MATLAB code for this purpose.

### Statistical analysis

Data was entered and statistically analyzed on the Statistical Package of Social Science Software program, version 25 (IBM SPSS Statistics for Windows, Version 25.0. Armonk, NY: IBM Corp.). Data was presented using mean and standard deviation for quantitative variables. Comparison between groups was conducted using independent sample t-test. P values ≤ 0.05 were considered statistically significant.

## Results

The comparison between sectional and circumferential matrices used in restoration of proximal contact and contour is outlined in Table 2.

**Fig. 3:**
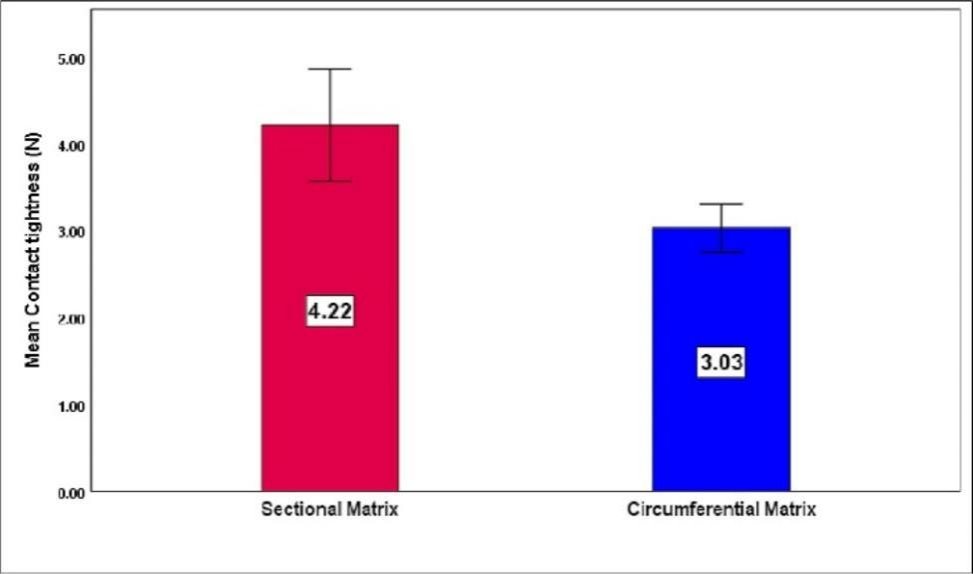
Mean contact tightness of sectional versus circumferential matrix

Sectional matrix group showed statistically significant tighter contacts compared to circumferential matrix group (4.22 ± 0.90 versus 3.03 ± 0.39 respectively) with p = 0.002 (Fig. 3).

**Fig. 4:**
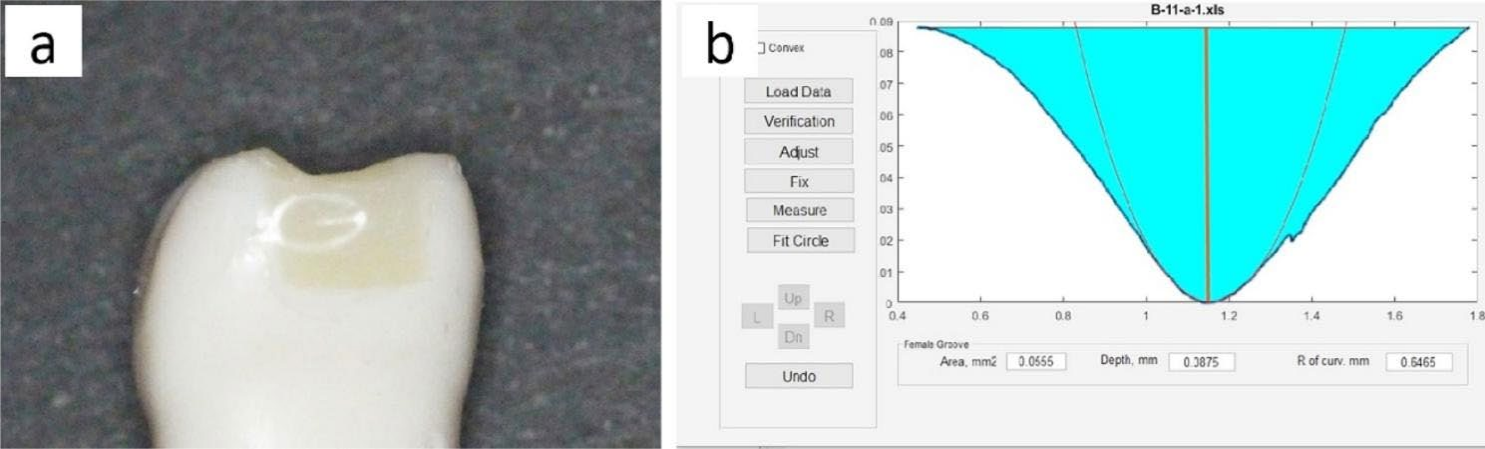
(**a**) concave profile at the contact area was found visually in restored tooth; (**b**) degree of concavity was evaluated by measuring the area, depth and radius of curvature

**Fig. 5:**
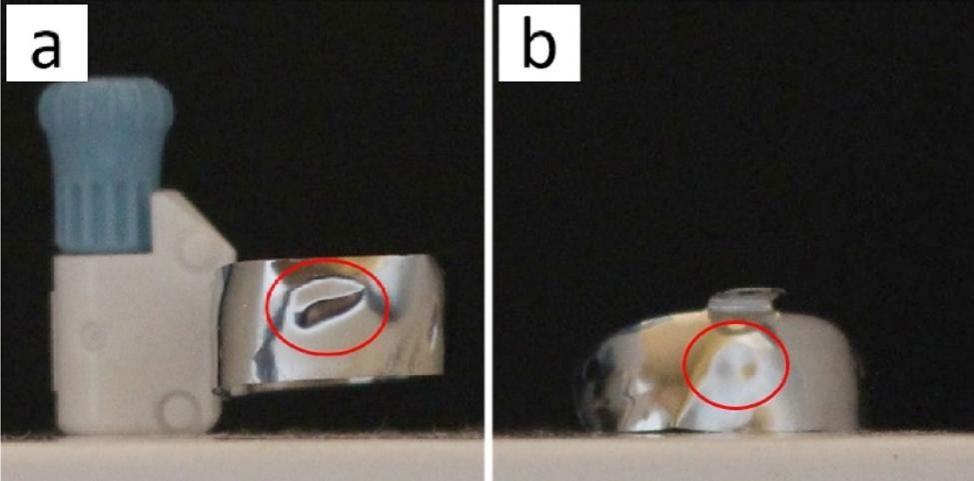
Distortion of matrix bands (red circles): (**a**) circumferential matrix, (**b**) sectional matrix

**Fig. 6:**
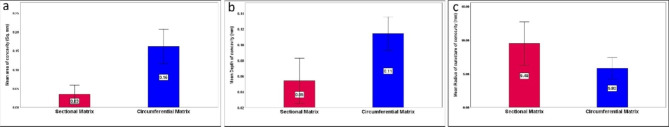
Mean of concavity of sectional versus circumferential matrix: (**a**) area, (**b**) depth, (**c**) radius of curvature

Both groups displayed concavity in restored teeth. Regarding the area and depth of concavity, the circumferential matrix group had statistically significant greater values (0.16 ± 0.06, 0.11 ± 0.03 respectively) than the sectional matrix group (0.03 ± 0.03, 0.05 ± 0.04 respectively) with p < 0.001, p = 0.001 respectively. Meanwhile, the radius of curvature of concavity was higher for sectional matrix group (9.48 ± 4.48) in comparison to circumferential matrix group (5.80 ± 2.24) with statistically significant difference (p = 0.036) as shown in Figs. 4, 5 and 6.

**Table 2:** Comparison between sectional and circumferential matrices used in restoration of proximal contact and contour

Parameter	Sectional Matrix	Circumferential Matrix	Mean Difference (95%CI)	P value
	Mean ± SD	Mean ± SD		
**Contact tightness (N)**	4.22 ± 0.90	3.03 ± 0.39	1.19 (0.52–1.86)	**0.002**
**Area of concavity (mm** ^**2**^ **)**	0.03 ± 0.03	0.16 ± 0.06	−0.13 (−0.18 − −0.08)	**< 0.001**
**Depth of concavity (mm)**	0.05 ± 0.04	0.11 ± 0.03	−0.06 (−0.09 − −0.03)	**0.001**
**Radius of curvature (mm)**	9.48 ± 4.48	5.80 ± 2.24	3.69 (0.27–7.10)	**0.036**

## Discussion

Tight proximal contact with proper contour play an important role in maintaining the integrity of dental arch and in periodontal health [4]. Proper reconstruction of proximal surface is based largely on the shape of matrix band and the accuracy of its placement [12]. Most sectional matrix systems consist of micro-thin pre-contoured sectional matrix, stainless steel ring with spring action, and elastic wedges. This system has been widely praised due to efficiency of sectional matrices in increasing proximal contact tightness and creating anatomical morphology [13], and; referred to as a gold standard [5].

Most circumferential matrices are very difficult to recreate anatomical emergence and result in inferior morphological contact with reduced contact tightness. Moreover, the matrix holder often limits access for wedge placement, which can have an impact on their efficacy [5].

Therefore, the current study investigated the new innovation of pre-contoured circumferential matrix system with integrated tightener to restore compound or complex class II resin composite restorations and compared it to the proven “gold standard”. During our study the circumferential matrix was time saving and easier to use due to its single component (without ring or holder) and easier technique of insertion (an integrated tightening mechanism).

The null hypothesis for this study was rejected as there was a significant difference between the matrix system types. In the present study, the mean contact tightness of sectional matrix was significantly higher than that of circumferential matrix. These results were in consistent with previous studies [3, 9, 14–17], which can be clarified by the wedging force of the accompanying separation ring and use of micro-thin contoured matrix band. This will cause tooth displacement and subsequently hampering to obtain a tight contact at treatment site. Meanwhile, the circumferential matrix showed lower contact tightness due to the fact that at two-surface cavities, it has to pass through the intact contact site of the tooth. Additionally, this system, in which no additional separation techniques were used, failed to produce tight contacts [5, 9].

Many clinical trials focused on contact tightness, although; achieving physiologic proximal contour is equally important in clinical practice [14]. A concavity in the proximal surface of the restoration is often not identifiable clinically and will be inaccessible to cleaning. This will harbour cariogenic biofilm with subsequent initiation and progression of caries in proximal surface of an unrestored adjacent tooth. Similar to contact tightness, the proximal contact morphology was correlated with the matrix-retainer systems [5, 18]. In the present study, the analysis of proximal contour revealed concavity at the contact area of composite restoration for both sectional and circumferential matrices. The results showed significantly higher concavity area and depth in circumferential matrix compared to sectional matrix. This was in line with Chuang et al. [3] who suggested the appearance of undesirable concavity in contact area when circumferential or sectional matrices were used [18]. Contrarily, their results showed that the concavity was more emphasized in the sectional matrix. This could be attributed to use of thicker circumferential flat matrix and Tofflemire retainer. In the current study, different circumferential matrix-retainer system as pre-contoured, 0.032 mm thin matrix with integrated tightener was used.

Although, micro-thin or dead-soft matrices with separation rings efficiently enhance the contact tightness compared to the circumferential matrix systems, the former matrices may deform and result in undesirable contact morphology [3]. Moreover, Bailey et al. [19] reported the occurrence of contact area concavity with the flexible metal matrices and separating ring technique tested in 86% of the restorations, whereas with the stiff metal matrix and novel ringless technique, this occurred in just 5%.

The presence of this concavity could be due to the distortion of matrix band. Distortion may occur at critical contact area during its placement, as this is the most bulbous part of the pre-contoured matrix. Therefore, the part that is most likely to be distorted by contact with the adjacent tooth during placement. Moreover, peripheral and central distortions often result from the tendency of the ring to tent the matrix. This happens by opening up gaps peripherally and forcing the contacting area against the adjacent tooth causing it to dimple [5]. Excessive packing force during manipulation of composite restoration with insufficient rigidity of matrix presented a plastic deformation of the matrix [3].

Additionally, based on the principle of “rope friction around pole”, friction force acting against the tension in the matrix strip can be applied [20]. The sectional matrix was fixed by an elastic spring tool acting on the two terminals of the matrix. Unlike circumferential matrix, where there are many contact points between the matrix and the tooth surface. Consequently, smaller friction force is produced that could not neutralize the tension force along the matrix. Thus, the sectional matrix seemed to be more stretched than in case of circumferential matrix. As a result, the sectional matrix is considered to provide more protection from deformation with less deficient restoration.

In our study we have focused on mesio-occlusal surface restoration which may be considered as a limitation. However, this was done for better standardization and exclusion of other variables as the comparator matrix system used was a sectional one. MOD cavities need to insert two rings with wedging that might affect the final results; Wirsching et al. [9] reported that the two rings and matrices placed simultaneously (mesially and distally) result in a separation effect in opposite direction and thus diminishing their effect. Further studies using different matrix systems could be of value.

## Conclusions

The use of separation ring with sectional matrix provides superior contact tightness compared to circumferential matrix. However, both matrix systems presented some deficiency regarding proximal contour of direct class II resin composite restoration.

We highly recommend using a more rigid matrix band to avoid deformation and using circumferential matrix with a ring or pre-wedging.

## Data Availability

All data included in this study are available from the corresponding author upon request.
